# Microsatellite Instability as a Risk Factor for Occult Lymph Node Metastasis in Early-Stage Endometrial Cancer: A Retrospective Multicenter Study

**DOI:** 10.3390/cancers17071162

**Published:** 2025-03-30

**Authors:** Carlo Ronsini, Stefano Restaino, Federico Paparcura, Giuseppe Vizzielli, Antonio Raffone, Mariano Catello Di Donna, Giuseppe Cucinella, Vito Chiantera, Pasquale De Franciscis

**Affiliations:** 1 Unit of Gynaecology and Obstetrics, Department of Woman, Child and General and Specialized Surgery, University of Campania “Luigi Vanvitelli”, 80168 Naples, Italy; antonio.raffone@unicampania.it (A.R.); pasquale.defranciscis@unicampania.it (P.D.F.); 2 Unit of Obstetrics and Gynecology, “Santa Maria della Misericordia” University Hospital, Azienda Sanitaria Universitaria Friuli Centrale, 33100 Udine, Italy; restaino.stefano@gmail.com (S.R.); paparcurafederico@gmail.com (F.P.); giuseppevizzielli@yahoo.it (G.V.); 3 Unit of Gynecologic Oncology, National Cancer Institute, IRCCS, Fondazione “G. Pascale”, 80131 Naples, Italy; marianocatellodidonna@gmail.com (M.C.D.D.); vito.chiantera@istitutotumori.na.it (V.C.); 4 Department of Health Promotion, Mother and Child Care, Internal Medicine and Medical Specialties (PROMISE), University of Palermo, 90133 Palermo, Italy

**Keywords:** microsatellite instability, endometrial cancer, occult lymph node metastasis, molecular classification, surgical staging

## Abstract

Objectives: This study investigates the association between microsatellite instability (MSI) and the risk of occult lymph node metastases (LNMs) in patients with early-stage endometrial cancer (EC) who showed no evidence of nodal involvement on preoperative imaging. Methods: A retrospective multicenter cohort study was conducted, including 237 patients with EC who underwent primary staging surgery between January 2022 and October 2024. The patients were stratified into two groups based on MSI status. The primary outcome was the prevalence of occult LNMs. Statistical analyses included univariate and multivariate logistic regression models, adjusting for potential confounders such as tumor grading and lymphovascular space invasion (LVSI). The significance of the models was assessed using the maximum likelihood method and Bayesian Information Criterion (BIC). Measures to reduce bias included blinding the data analyst, standardization of histopathological evaluation, and exclusion of patients with genetic conditions predisposing to MSI. Results: The MSI group had a significantly higher incidence of occult LNMs compared to the MSS group (19% vs. 6.7%, *p* = 0.005). The multivariate analysis confirmed MSI as an independent risk factor for LNMs (OR = 1.105, 95% CI 1.016–1.202, *p* = 0.020). The sub-analysis showed that loss of *MLH1/PMS2* or both *MLH1/PMS2* and *MSH2/MSH6* heterodimers further increased LNMs risk, independently from other risk factors. Conclusions: MSI is independently associated with a higher risk of occult LNMs in early-stage EC, suggesting a potential role for MSI profiling in refining lymph node staging strategies. Future prospective studies should assess the prognostic impact of this association and its implications for surgical decision-making.

## Simple Summary

Endometrial cancer is one of the most common gynecological cancers, and its spread to lymph nodes significantly affects treatment decisions and patient outcomes. Current imaging techniques, such as CT and MRI, often fail to detect small or hidden metastases, leading to uncertainty in staging and treatment planning. This study investigates whether microsatellite instability, a genetic alteration found in some endometrial cancers, is linked to a higher risk of hidden lymph node metastases. By analyzing data from 237 patients, we found that tumors with microsatellite instability are more likely to have occult lymph node involvement. These findings suggest that testing for microsatellite instability could help doctors identify high-risk patients who may benefit from more extensive lymph node evaluation.

## 1. Introduction

Endometrial cancer (EC) is the most common gynecological cancer in high-income countries, with an increasing incidence over the past few decades [1]. The presence of lymph node metastases (LNMs) is a major prognostic factor in EC and an indication for adjuvant treatment [2]. The prognosis is also influenced by several other factors, including the extent of myometrial invasion, lympho-vascular invasion (LVSI), grading, adnexal involvement, peritoneal dissemination, and distant metastasis. The pelvic and paraaortic lymph nodes are the most common sites for LNMs [3,4]. There is an association between preoperative radiological testing and identifying patients at lower risk for LNMs rather than at greater risk. However, ultrasound [5], CT [6], and MRI [7] are not always effective in identifying patients with para-aortic or pelvic metastases. An analysis by the Gynecology Oncology Group found that 22% of patients with clinically apparent stage I EC had pelvic (9%) and/or para-aortic (6%) LNMs [8]. Therefore, the risk of LNMs in EC cannot be objectively and reproducibly assessed before surgery.

Nowadays, the Proactive Molecular Risk Classifier for Endometrial Cancer (ProMisE) is being used in clinical settings [9] as a result of the discovery of the four molecular subtypes of EC by The Cancer Genome Atlas (TCGA) in 2013 [10]. Molecular and genomic profiling has been a significant innovation in diagnosing and treating endometrial cancer. According to ProMisE, EC can be classified into four molecular subtypes based on a combination of targeted sequencing for *POLE* exonuclease domain mutations and immunohistochemistry (IHC) to determine mismatch repair proteins (MMR) and p53 status: POLEmut, mismatch repair deficient (MMRd), p53abn, and NSMP (no specific molecular profile). An immunohistochemical deficiency of MMR characterizes the MMRd group, which has an intermediate prognosis, high mutational load, and microsatellite instability (MSI) [11]. There are four MMR proteins involved, and they are organized into two heterodimers: *MLH1/PMS2* and *MSH2/MSH6*. Loss of function of even one of these heterodimers results in an MSI condition. Accordingly, within the MMRd group, there will be ECs with a loss of both heterodimers or only one of them.

Despite the widespread use of CT, PET-CT, and magnetic resonance imaging (MRI) in the preoperative evaluation of lymph node involvement in patients with endometrial cancer, these tools have limited sensitivity in detecting microscopic metastases or occult micrometastases in nonsignificantly enlarged lymph nodes. Previous studies have shown that PET-CT may have less than 70% sensitivity for detecting small lymph node metastases, and CT and MRI are even less reliable. These limitations underscore the need to incorporate molecular biomarkers, such as the MSI status, in preoperative staging to improve risk stratification and optimize the selection of lymphadenectomy candidate patients [12].

In a retrospective study, Jamieson et al. identified 172 patients undergoing sentinel node mapping followed by lymphadenectomy. The authors observed a correlation between molecular classification and LNMs (p53 abnormal 44.8%; MMRd 14.9%; POLE mutated 14.2%; NSMP 10.8%) [13]. In a prospective study of 223 patients with apparent early-stage endometrial cancer undergoing sentinel node mapping, Bogani et al. provided evidence that deep myometrial invasion and LVSI are associated with LNMs. However, no association was found between the molecular classes and the risk of having LNMs [14]. Molecular classification could be useful in identifying patients who are at higher risk for LNMs. However, further studies must be conducted to validate these data. Incorporating molecular classification into the decision-making process for nodal dissection will improve surgical strategies and lead to a more personalized approach to endometrial cancer care.

### Objective

This retrospective study investigates whether occult LNMs and Micro-Satellite Instability (MSI) are related in patients with apparent early-stage EC with no evidence of LNMs in preoperative CT/Total body PET-CT. As a retrospective observational cohort study, this work compares patients with Micro-Satellite Stability (MSS) and MSI and the incidence of LNMs. Thus, this evidence could strongly underline the importance of expanding the evaluation of the MSI status in patients with EC.

## 2. Materials and Methods

### 2.1. Study Design

We conducted a retrospective observational multicenter cohort study with secondary data from a clinical database, selecting a total of 237 patients with EC who underwent primary staging surgery at the Gynaecologic oncology unit of the University of Campania Luigi Vanvitelli, Naples, Italy, and the Unit of Obstetrics and Gynecology, “Santa Maria della Misericordia” University Hospital, Udine, Italy. This study was conducted using the STROBE statement for observational studies [15]. A dedicated consent form for anonymous data processing and a study-specific consent form was required for all patients treated in the study. According to the regulations in force in the state where the study took place, an IRB from the internal ethical board was acquired and registered on 24 November 2022 with number 0035563/I (Comitato etico università degli studi “Luigi Vanvitelli”).

The study’s primary outcome was to assess the difference in the prevalence of occult LNMs in MSI and MSS ECs with negative preoperative CT/PET-CT examination.

We also conducted a sub-analysis of the types of defective heterodimers that led to a deficit in mismatch repair (MMRd).

### 2.2. Setting

Between January 2022 and October 2024, all patients treated for EC at the Gynaecologic oncology unit of the University of Campania “Luigi Vanvitelli”, Naples, Italy, and the Unit of Obstetrics and Gynecology, “Santa Maria della Misericordia” University Hospital, Udine, Italy, who met the inclusion criteria were enrolled in this retrospective observational study. All included patients had a total hysterectomy with bilateral salpingo-oophorectomy and bilateral pelvic node mapping (by sentinel lymph node SLN or lymphadenectomy) and had been subjected to a preoperative CT/Total body PET-CT with no evidence of LNMs in the 30 days previous surgery, revised by two independent blinded expert radiologists. The patients were stratified based on molecular profile in two groups: MSI and MSS. All the enrolled patients had histological diagnoses of EC and at least bilateral pelvic SLN exploration. No follow-up was needed for the desired data.

### 2.3. Participants

The inclusion criteria for participation in the study were: Clinical FIGO Stage I EC; complete pathological information regarding EC; complete information regarding the clinical status at the time of diagnosis based on a preoperative CT scan or a total body PET scan whose results are negative for LNMs performed not more than 30 days before surgery and revised by two independent blinded expert radiologists; complete information regarding the expression of genes *MLH1*, *PMS2*, *MSH2*, and *MSH6*; patients who underwent primary staging surgery for bilateral lymph node study; patients who received SLN mapping or lymphadenectomy, bilaterally, and studied for metastasis by ultrastaging; and patients older than 18 years old.

The exclusion criteria were: conditions associated with genetic susceptibility to MSI, including Lynch syndrome diagnosis; anatomopathological information obtained from endometrial biopsy rather than analysis of the whole endometrium; patients diagnosed with previous cancers in the last 5 years; patients undergoing previous chemotherapy; and patients undergoing previous pelvic radiotherapy.

### 2.4. Variables

The variables examined were the microsatellite stability status detected by immunohistochemical profiling (IHC) for MMRp (*MLH1*, *PMS2*, *MSH2*, and *MSH6*), which were then grouped into 2 classes, MSI and MSS; the body mass index (BMI) as a continuous variable in kg/m^2^; age, expressed as a continuous variable in years; ethnicity, considered as a categorical variable; the histotype of EC, understood as a histological diagnosis of certainty and considered as a categorical variable; grading, assessed from 1 to 3, based on cytoarchitectonic alteration, judged separately by 2 expert pathologists, and considered as an ordinal variable; LVSI, based on the absence or presence of <3 tumor cells per each LVS or more than 3 and stratified, respectively, into “negative”, “focal”, and “diffuse” and considered as an ordinal variable; myometrial infiltration of cancer, based on the millimeter of deepness in the myometrium into the thickness of the entire myometrial wall, divided into “no infiltration”, “infiltration < 50%”, and “infiltration ≥ 50%”, and considered as an ordinal variable. The p53 status was expressed as a dichotomous variable and considered mutated in case of null expression or hyperexpression of the p53 oncoprotein. Otherwise, it has been considered wild type (wt). The outcome variable of interest was the prevalence of LNM, which was described as a dichotomous variable (positive or negative). The positivity was considered for at least one micrometastasis in at least one node. Every node has been studied by ultrastaging immunochemistry. The ultrastaging result was determined by the judgment of 2 expert pathologists. It could be negative in the absence of neoplastic cells, macrometastasis for lesions ≥ 2 mm, micrometastasis for lesions < 2 mm, and isolated tumor cells (ITC) when it was <0.2 mm [16]. In this case, the lymph node was considered negative for study purposes. Finally, the number of lymph nodes excised and the number of positive lymph nodes was reported as a continuous variable.

### 2.5. Laboratory

Immunohistochemical profiling (IHC) for MMRP was performed using the antibodies *MLH1* (clone M1 Ventana), *PMS2* (clone EPR3947 Cell Marque), *MSH2* (clone G219-1129 Cell Marque), and *MSH6* (clone 44 Ventana). Normal expression of MMRP in tumor cells is represented by strong nuclear immunoreactivity for all four markers, with staining of comparable intensity to that of the internal control, represented by the normal intestinal mucosa, stromal and lymphoid cells, or the appendix [17]. This result indicates a ‘stable state’ of microsatellites (MSS). A two-step analysis was performed because *MLH1-PMS2* and *MSH2-MSH6* are a couple of heterodimeric genes [18]. The evaluation started with *PMS2* and *MSH6* expression. In the case of loss of expression, *MLH1* and *MSH2* were subsequently examined to distinguish between isolated and concomitant loss. Tumors were considered MSI if at least one of the four MMR proteins (*MSH2*, *MSH6*, *PMS2*, or *MLH1*) had a loss of expression. All MSI patients were subjected to germline genetic testing by next-generation sequencing (NGS) to confirm Lynch syndrome diagnosis. In case of confirmation, the patients were excluded from the study.

### 2.6. Statistical Analysis

The null hypothesis of our study was that there was no difference in the prevalence of LNMs in the 2 different classes, MSI and MSS (H0: π_1_ = π_2_).

Previously, all variables were analyzed using histograms and compared with parametric distributions and non-parametric distributions. Due to their non-parametric distribution, the continuous variables were expressed as medians and interquartile ranges, and the Wilcoxon signed-rank test was used to compare them [19]. Dichotomous and Ordinal variables were expressed as absolute numbers and percentages and compared using Fisher’s exact test [20] or a Chi-squared test, according to sample numerosity.

The weights of the individual values on the dependent variables (occult LNM) were calculated as a logarithmic regression and a multivariate regression analysis [21]. The significance of the model used was assessed using the maximum likelihood method [22].

To determine whether the sample was adequate to detect a statistically significant difference in LNM risk between the MSI and MSS groups, an a posteriori power analysis based on the Chi-square test for independent proportions was performed. With a significance level α = 0.05 and a true difference in the incidence of lymph node metastasis between the two groups (19% vs. 6.7%), the resulting statistical power (1 − β) was 85%, indicating a high probability of detecting a true association. The statistical significance level was set at 0.05, and all statistical investigations were performed using R software version 2024.4.2 and R Studio version 2023.12.1 + 402.

### 2.7. Risk of Bias

All variables were combined in multivariate regression studies to minimize confounders.

As a result, the final individual models were compared using adjusted R2 and Bayesian Information Criteria (BIC). The best model was selected based on the lowest expressed value of BIC [23]. Data analysis was conducted first by CR and then by Blinding by MCDD, who was unaware of the study’s objective. No missing data were present in the outcomes of interest.

## 3. Results

Out of 251 potentially recruitable patients, because of exclusion criteria, 237 patients with apparent early-stage EC treated for endometrial cancer between January 2022 and October 2024 were enrolled in this retrospective observational study. The sample was stratified by microsatellite stability in MSI (57, 24%) and MSS (180, 76%) groups. The two groups showed a statistically significant difference only in tumor grading (MSI G3 39%, and MSS 27%; *p* = 0.001) and myometrial infiltration (≥50% of the myometrium in 47% of MSI and in 24% of MSS, *p* < 0.001). Age, BMI, ethnicity, histotype, LVSI, and p53 status did not show any significant differences. The characteristics of the population are summarized in Table 1.

### 3.1. Outcomes

The study’s primary outcome was to assess the difference in the prevalence of occult LNMs in MSI and MSS ECs with negative preoperative CT/PET-CT examination. We had no missing data for the outcomes of interest.

The MSI group showed a statistically significantly higher number of occult LNMs (19% in MSI vs. 6.7% in MSS, *p* = 0.005), as reported in Table 2.

We constructed a logarithmic regression model to test the correlation between occult LNMs and MSI.

The univariate logarithmic model was constructed with occult LNMs as the dependent variable and the MSI status as the independent variable. MSI showed a statistically significant positive correlation with occult LNMs with an OR of 1.134 (estimate 0.126, std error 0.044; 95% CI 1.04–1.24; *p*-value 0.005). The data are reported in Table 3.

We also constructed multivariate regression models. All the combinations of significantly different variables between the two groups of interest were used to propose different models. Based on the lowest BIC, the best multivariate analysis model combines Grading and LVSI status as confounders. Even in the multivariate model, MSI retained a statistically significant association with LNM, with an OR of 1.105 (estimate 0.006, std error 0.043; 95% CI 1.02–1.20; *p*-value 0.020). Diffuse LVSIs have also been shown to play a role in LNMs with an OR of 1.240 (estimate 0.216, std error 0.046; 95% CI 1.13–1.36; *p*-value 0.001) (Table 4).

### 3.2. Sub-Analysis for Heterodimeric Genes

We conducted a sub-analysis based on the loss of specific heterodimers underlying the MSI: loss of *MLH1-PMS2* (38, 66.7%), MSH2-MSH6 (11, 19.3%), or both heterodimers (8, 14%). The probability of having LNMs was statistically significantly higher in case of loss of function of both heterodimers and just the couple *MLH1/PMS2* (intact expression 6.6%, both 38%, *MLH1/PMS2* 21%, *MSH2/MSH6* 0%; *p* = 0.002). The data are summarized in Table 5.

A second univariate logarithmic regression model was constructed with occult LNMs as the dependent variable and loss of heterodimers *MLH1-PMS2*, loss of *MSH2-MSH6*, or loss of both heterodimers as independent variables. The strongest association was recorded in the case of loss of both the heterodimers (OR 1.36, estimate 0.309, std error 0.104; 95% CI 1.11–1.67; *p*-value 0.003), followed by the loss of *MLH1/PMS2* (OR 1.16, estimate 0.150, std error 0.052; 95% CI 1.11–1.67; *p*-value 0.004). No statistically significant association was found between LNMs and isolated loss of *MSH2/MSH6*. The data are summarized in Table 6.

We also constructed multivariate regression models for each proposed univariate. All the combinations of significantly different variables between the two groups of interest were used to propose different models. Based on the lowest BIC, the best multivariate analysis model, also in this case, combines as confounders Grading and LVSI status. In both multivariate models, the significance of the correlation with loss of *MLH1/PMS2* or both heterodimers was preserved (Table 7).

## 4. Discussion

### 4.1. Interpretation of the Results

Our study shows a correlation between MSI and LNMs. Not only are LNMs more frequent in MMRd patients, but there is a direct correlation between microsatellite instability and the risk of having LNMs. This risk is maintained even without additional known factors for LNMs such as LVSI [24]. Moreover, the sub-analysis conducted made it possible to identify the heterodimers whose loss of function most affects the risk of LNMs. As reported in the results, the *MLH1/PMS2* heterodimer loss has a greater weight than the *MSH2/MSH6* pair. This finding is also supported by the evidence that in the case of the loss of both heterodimers, the risk is increased compared with the loss of *MSH2/MSH6* alone. Finally, a key to interpreting the results is that 19% of MSI patients, even in the absence of instrumental suspicion, showed occult LNMs. This means that 14% of MSI patients have non-preoperatively suspected lymph node metastasis. These data prove that MSI is an independent risk factor for developing LNMs in EC. The molecular mechanisms by which this occurs need to be further verified with studies focused on this question.

### 4.2. Comparison with the Existing Literature

Microsatellite instability represents an intermediate prognosis condition in endometrial carcinomas [9]. Moreover, the MSI condition alone is more likely to be associated with conditions of greater prognostic impact. For example, and as also reported in our sample, deep myometrial infiltration is more frequent in the MSI group [25]. Similarly, MSI seems to correlate more easily with high grading and LVSI [26]. All these conditions paint a particularly connected picture in which the MMRd status plays into processes of tumor progression, leading to prognostic impact. In such a scenario, the evidence arising from our study, in which LNMs are more frequent in MSI ECs, is not surprising. One hypothesis supporting these data comes from MSI tumors’ known more remarkable genomic plasticity, facilitating their adaptation and progression [27]. In addition, the MMRd status leads to strong activation of epithelial–mesenchymal processes (EMT), promoting invasion and dissemination [28].

### 4.3. Clinical Implication

The multivariate analysis proves a correlation between MSI and the possibility of occult lymph node metastasis. Although this finding is less influential than other parameters, such as LVSI positivity, awareness of this correlation could affect clinical choices. Lymph node staging is a necessary step in endometrial cancer [29]. Still, it is often bypassed in malpractice for patients considered at low risk of lymph node metastasis or in patients with high surgical morbidity. Our study may help reevaluate these decisions in light of the increased risk for MSI patients. In addition, a second point of interest might be to evaluate the adequacy of the SLN in this type of patient. The SLN appears to be highly reliable in cases of negativity [30], but this evidence comes from studies that do not consider the unstable state of the subjects’ microsatellites instability. Our study, by its nature, does not offer a prognostic view of this lymph node positivity. Still, it might be of interest in future considerations to build investigation models that assess the safety of SLN in patients at specific high risk of lymph node metastasis, such as diffuse LVSI and MSI. Finally, the increased risk of occult metastasis should also be a parameter to be considered in cases of Fertility Sparing Treatments (FST). These are possible only for patients with low grading and no myometrial infiltration [31]. Any lymph node positivity contraindicates any FST. However, lymph node negativity is attributed to imaging. In patients at high risk of occult metastasis, this principle could be revised in light of these findings.

### 4.4. Strengths and Limitations

Our study inherits several limitations from its retrospective design. First, the lymph node staging examined is pelvic. Following the care criteria of the recruiting centers, lumboaortic lymphadenectomy is reserved only for cases of macroscopic evidence of lymph node compromise, and, therefore, since these are patients enrolled in the absence of suspected lymph node metastasis, none of the enrolled patients received a lumboaortic lymphadenectomy. Second, a limitation is the variability of preoperative staging examinations, including CT and PET-CT. However, this study has a sound methodological construction, with stringent inclusion and exclusion criteria. In addition, the strategies implemented to minimize bias improved the reliability of the data obtained, which gave us strong statistical evidence. It should be a starting point for constructing prospective and more standardized studies in surgical technique and staging imaging and investigations into the prognostic significance of lymphadenectomy in this group of patients. By the same principle, having been excluded from the study, these data do not apply to patients whose MSI depended on genetic syndromes such as Lynch syndrome. Future studies should evaluate whether the association between MSI and LNM risk differs in patients with Lynch syndrome, given their distinct molecular backgrounds.

Another limitation of our study is that the MMR system consists of seven genes (*MLH1, MLH3, MSH2, MSH3, MSH6, PMS1, and PMS2*), yet we only assessed the protein expression of *MLH1, MSH2, MSH6, and PMS2*. The significance of other MMR genes in developing the MSI phenotype in endometrial cancer has been documented in the literature. Notably, EEC G3 harbors the highest frequency of MMR mutations among endometrial cancers and could be prioritized for MMR mutation screening, as they exhibit a higher prevalence of these alterations than EEC G1 and G2. In addition to *MLH1, MSH2, MSH6, PMS2, and EPCAM* mutations, screening for *MLH3, MSH3, and PMS1* mutations may benefit newly diagnosed endometrial carcinoma patients [32].

## 5. Conclusions

Our study shows a correlation between MSI and occult lymph node metastasis in EC. This association appears independent of other histopathological parameters, such as diffuse LVSI involvement and grading 3. The evidence that has arisen from this study may provide the basis for future prospective analyses also aimed at estimating the prognostic impact of this association.

## Figures and Tables

**Table 1:** Patients’ characteristics.

Characteristic	MSS, N = 180 ^1^	MSI, N = 57 ^1^	*p*-Value
Age	62 (56; 71)	64 (59; 71)	0.142
BMI	29 (25; 32)	30 (27; 34)	0.166
Missing	1	0	
Ethnicity			>0.999
Caucasian	178 (99%)	57 (100%)	
Hispanic	1 (0.6%)	0 (0%)	
Indian	1 (0.6%)	0 (0%)	
Histology			0.369
Endometrioid	155 (86%)	52 (91%)	
Serous	25 (14%)	5 (8.8%)	
Grading			**0.001**
1	66 (37%)	7 (12%)	
2	66 (37%)	28 (49%)	
3	48 (27%)	22 (39%)	
LVSI			0.296
Negative	123 (69%)	35 (61%)	
Focal	16 (9.0%)	9 (16%)	
Diffuse	39 (22%)	13 (23%)	
Missing	2	0	
Myometrial Invasion			**<0.001**
No Infiltration	2 (1.1%)	2 (3.5%)	
<50%	134 (74%)	28 (49%)	
≥50%	44 (24%)	27 (47%)	
p53			0.646
mut	21 (12%)	8 (14%)	
wt	159 (88%)	49 (86%)	

^1^ Median (Q1–Q3); n (%); LVSI, Lymphvascular Space Invasion; Mut, mutated; Wt, wild type.

**Table 2:** Outcomes.

Characteristic	MSS, N = 180	MSI, N = 57	*p*-Value
Lymph node, n (%)			**0.005**
Negative	168, (93%)	46, (81%)	
Positive	12, (6.7%)	11, (19%)	

**Table 3:** Logit regression MSI.

Variable	Estimate	Std. Error	z Value	*p*-Value	conf.low	conf.high	Odds Ratio	OR 95% CI
MSI	0.126	0.044	2.843	**0.005**	0.039	0.213	1.134	1.04–1.237

**Table 4:** Multivariate regression MSI.

Variable	Estimate	Std. Error	z Value	*p*-Value	conf.low	conf.high	Odds Ratio	OR 95% CI
MSI	0.100	0.043	2.344	**0.020**	0.016	0.184	1.105	1.016–1.202
Grading 3	0.020	0.025	0.790	0.430	−0.029	0.068	1.020	0.971–1.07
LVSI Diffuse	0.216	0.046	4.676	**0.001**	0.126	0.307	1.24	1.134–1.359

**Table 5:** Outcomes for the heterodimers.

Characteristic	Intact Nuclear Expression, n = 180 ^1^	Loss of Both Heterodimers, n = 8 ^1^	Loss of *MLH1/PMS2*, n = 38 ^1^	Loss of *MSH2/MSH6*, n = 11 ^1^	*p*-Value ^2^
Lymphnode					**0.002**
Negative	168 (93.3%)	5 (63%)	30 (79%)	11 (100%)	
Positive	12 (6.6%)	3 (38%)	8 (21%)	0 (0%)	

^1^ n (%); ^2^ Fisher’s exact test.

**Table 6:** Logit regression heterodimers.

Variable	Estimate	Std. Error	z Value	*p*-Value	conf.low	conf.high	Odds Ratio	OR 95% CI
Loss of both heterodimers	0.309	0.104	2.965	**0.003**	0.105	0.513	1.362	1.111–1.67
Loss of *MLH1/PMS2*	0.150	0.052	2.883	**0.004**	0.048	0.252	1.162	1.049–1.287
Loss of *MSH2/MSH6*	−0.066	0.089	−0.741	0.460	−0.242	0.109	0.936	0.785–1.115

**Table 7:** Multivariate regression heterodimers.

Variable	Estimate	Std. Error	t Value	*p*-Value	conf.low	conf.high	OR	OR 95% CI
Loss of both heterodimers	0.286	0.098	2.920	**0.004**	0.094	0.478	1.331	1.099–1.613
Loss of *MLH1/PMS2*	0.113	0.050	2.271	**0.024**	0.015	0.211	1.120	1.015–1.235
Loss of *MSH2/MSH6*	−0.065	0.084	−0.777	0.438	−0.230	0.100	0.937	0.795–1.105
Grading 3	0.020	0.024	0.821	0.412	−0.028	0.068	1.020	0.972–1.07
LVSI Diffuse	0.211	0.046	4.634	**0.001**	0.122	0.301	1.235	1.13–1.351

## Data Availability

All data and the methodological process for their calculation can be supplied under explicit request to the corresponding author and provided as an ‘.R’ file.

## Abbreviations

The following abbreviations are used in this manuscript:

ART	Assisted Reproductive Technology
BIC	Bayesian Information Criterion
BMI	Body Mass Index
CI	Confidence Interval
CT	Computed Tomography
EC	Endometrial Cancer
EMT	Epithelial-Mesenchymal Transition
FST	Fertility-Sparing Treatment
IHC	Immunohistochemistry
ITC	Isolated Tumor Cells
LNM	Lymph Node Metastasis
LVSI	Lymphovascular Space Invasion
MRI	Magnetic Resonance Imaging
MMR	Mismatch Repair
MMRd	Mismatch Repair Deficient
MSI	Microsatellite Instability
MSS	Microsatellite Stability
NSMP	No Specific Molecular Profile
OR	Odds Ratio
PET-CT	Positron Emission Tomography—Computed Tomography
POLEmut	Polymerase Epsilon Mutant
ProMisE	Proactive Molecular Risk Classifier for Endometrial Cancer
SLN	Sentinel Lymph Node
STROBE	Strengthening the Reporting of Observational Studies in Epidemiology
TCGA	The Cancer Genome Atlas
wt	Wild Type
